# Supplementary material for: Structural modeling for Oxford histological classifications of immunoglobulin A nephropathy
Source: PLoS One. 2022 Sep 9;17(9):e0268731. doi: 10.1371/journal.pone.0268731 (PMC9462802; doi:10.1371/journal.pone.0268731)
Supplement: S1 File — (DOCX) [file pone.0268731.s001.docx]

**Supplementary Material 1: A flowchart for analyzing the process**

Step 1. Selection of histological, clinical, and treatment variables

Step 2. Linear regression analysis among non-selected histological, clinical, and treatment variables

2−1: M, E, S, T, and C to SReGFR0c or MAPc01(path d in Fig. 1)

2−2: M, E, S, T, C, SReGFR0c,and MAP c01 to UPE0c(path d in Fig. 1)

2−3: M, E, S, T, C, UPE0c, SReGFR0c, and MAP c01 to ST (paths e and f in Fig. 1)

2−4: M, E, S, T, C, UPE0c, SReGFR0c, and MAP c01 to RASB (path e and f in Fig. 1)

2−5: M, E, S, T, C, UPE0c, SReGFR0c,MAP c01, ST, and RASB to SLOPE (paths a, b, and c in Fig. 1)

Step 3. After eliminating nonsignificant paths (*P* > 0.05), significant paths for the histological, clinical, and treatment variables were chosen, and they are displayed in Table 2.

| **SLOPE** | SC | SE | z | P > z | 95% Confidence Interval | |
| --- | --- | --- | --- | --- | --- | --- |
| MAPc01 | −0.052 | 0.034 | −1.510 | 0.130 | −0.120 | 0.015 |
| SReGFR0c | −0.279 | 0.041 | −6.750 | 0.000 | −0.359 | −0.198 |
| UPE0c | −0.067 | 0.034 | −2.000 | 0.046 | −0.133 | −0.001 |
| ST | 0.115 | 0.033 | 3.420 | 0.001 | 0.049 | 0.180 |
| RASB | −0.034 | 0.035 | −0.990 | 0.324 | −0.103 | 0.034 |
| M | 0.000 | 0.034 | −0.010 | 0.994 | −0.068 | 0.067 |
| S | −0.001 | 0.034 | −0.030 | 0.978 | −0.067 | 0.066 |
| E | 0.015 | 0.035 | 0.420 | 0.676 | −0.054 | 0.083 |
| T1orT2 | −0.135 | 0.040 | −3.380 | 0.001 | −0.213 | −0.056 |
| C1orC2 | 0.010 | 0.036 | 0.280 | 0.781 | −0.061 | 0.081 |
| _cons | −0.213 | 0.086 | −2.490 | 0.013 | −0.381 | −0.046 |
| **MAPc01** |  |  |  |  |  |  |
| M | 0.011 | 0.033 | 0.340 | 0.731 | −0.054 | 0.077 |
| S | 0.100 | 0.033 | 3.030 | 0.002 | 0.035 | 0.164 |
| E | 0.027 | 0.034 | 0.780 | 0.433 | −0.041 | 0.095 |
| T1orT2 | 0.238 | 0.032 | 7.520 | 0.000 | 0.176 | 0.300 |
| C1orC2 | −0.054 | 0.035 | −1.530 | 0.126 | −0.123 | 0.015 |
| _cons | 0.642 | 0.068 | 9.400 | 0.000 | 0.508 | 0.776 |
| **SReGFR0c** |  |  |  |  |  |  |
| M | −0.034 | 0.028 | −1.210 | 0.226 | −0.088 | 0.021 |
| S | −0.094 | 0.027 | −3.430 | 0.001 | −0.147 | −0.040 |
| E | −0.001 | 0.029 | −0.030 | 0.980 | −0.057 | 0.055 |
| T1orT2 | −0.571 | 0.021 | −26.690 | 0.000 | −0.613 | −0.529 |
| C1orC2 | 0.022 | 0.029 | 0.760 | 0.447 | −0.035 | 0.080 |
| _cons | 0.446 | 0.051 | 8.670 | 0.000 | 0.345 | 0.547 |
| **UPE0c** |  |  |  |  |  |  |
| MAPc01 | 0.019 | 0.033 | 0.570 | 0.566 | −0.046 | 0.083 |
| SReGFR0c | −0.198 | 0.039 | −5.070 | 0.000 | −0.275 | −0.122 |
| M | 0.190 | 0.032 | 5.950 | 0.000 | 0.128 | 0.253 |
| S | 0.000 | 0.033 | −0.010 | 0.990 | −0.064 | 0.064 |
| E | 0.025 | 0.034 | 0.730 | 0.463 | −0.041 | 0.091 |
| T1orT2 | 0.001 | 0.039 | 0.030 | 0.980 | −0.076 | 0.078 |
| C1orC2 | 0.089 | 0.035 | 2.580 | 0.010 | 0.021 | 0.157 |
| _cons | −0.227 | 0.069 | −3.260 | 0.001 | −0.363 | −0.091 |
| **ST** |  |  |  |  |  |  |
| MAPc01 | −0.069 | 0.033 | −2.110 | 0.035 | −0.133 | −0.005 |
| SReGFR0c | 0.096 | 0.040 | 2.420 | 0.016 | 0.018 | 0.175 |
| UPE0c | 0.105 | 0.032 | 3.260 | 0.001 | 0.042 | 0.168 |
| M | 0.020 | 0.033 | 0.610 | 0.543 | −0.045 | 0.085 |
| S | 0.126 | 0.032 | 3.890 | 0.000 | 0.062 | 0.189 |
| E | 0.114 | 0.033 | 3.410 | 0.001 | 0.048 | 0.179 |
| T1orT2 | −0.026 | 0.039 | −0.670 | 0.500 | −0.102 | 0.050 |
| C1orC2 | 0.178 | 0.034 | 5.230 | 0.000 | 0.112 | 0.245 |
| _cons | 0.962 | 0.078 | 12.370 | 0.000 | 0.809 | 1.114 |
| **RASB** |  |  |  |  |  |  |
| MAPc01 | 0.199 | 0.031 | 6.340 | 0.000 | 0.137 | 0.260 |
| SReGFR0c | −0.266 | 0.038 | −7.000 | 0.000 | −0.340 | −0.191 |
| UPE0c | 0.076 | 0.031 | 2.460 | 0.014 | 0.015 | 0.137 |
| M | 0.028 | 0.032 | 0.880 | 0.380 | −0.035 | 0.091 |
| S | 0.050 | 0.031 | 1.590 | 0.112 | −0.012 | 0.111 |
| E | 0.040 | 0.032 | 1.230 | 0.218 | −0.024 | 0.103 |
| T1orT2 | 0.040 | 0.037 | 1.060 | 0.291 | −0.034 | 0.113 |
| C1orC2 | −0.025 | 0.033 | −0.760 | 0.449 | −0.091 | 0.040 |
| _cons | 0.851 | 0.073 | 11.640 | 0.000 | 0.707 | 0.994 |
|  |  |  |  |  |  |  |
| SC, standardized coefficient; SE, standard error; 281 T, tubular atrophy/interstitial fibrosis;  M, mesangial hypercellularity; C, active crescent; S, segmental glomerulosclerosis;  E,endocapillary hypercellularity; ST, steroid therapy including tonsillectomy;  RASB, renin-angiotensin system blocker; UPE0c, centralized baseline urine protein excretion;  SReGFR0c, centralized square root baseline eGFR; MAPc01, centralized dichotomized baseline means arterial pressure; cons: constant | | | | | | |
|  | | | | | | |
|  | | | | | | |
|  | | | | | | |
|  | | | | | | |

Step 4. In Fig. 2, it is shown that selected significant correlations exist between changes in estimated glomerular filtration rate (SLOPE) and histological, clinical, and treatment variables. After eliminating nonsignificant paths (*P* > 0.05), SEM was performed again to confirm a model that suited the data well. An appropriate fitting model was proven using population error (root mean square error of approximation [RMSEA] < 0.05 with 90% CI), baseline comparison (comparative fit index [CFI] > 0.90), and residual size (standardized root mean square residual [SRMSR] < 0.05).

| **Likelihood ratio** |  | |
| --- | --- | --- |
| chi2_ms (25) | 89.63 | Model vs. saturated |
| p > chi^2^ | 0.00 |  |
| chi^2^_bs (45) | 1015.04 | Baseline vs. saturated |
| p > chi^2^ | 0.00 |  |
| **Population error** |  | |
| RMSEA | 0.05 |  |
| 90% CI, lower bound | 0.04 |  |
| upper bound | 0.06 |  |
| Probability RMSEA ≤0.05 | 0.35 |  |
| **Information criteria** |  | |
| AIC | 18063.63 |  |
| BIC | 18218.90 |  |
| **Baseline comparison** |  | |
| CFI | 0.93 |  |
| TLI | 0.88 |  |
| **Size of residuals** |  | |
| SRMR | 0.03 |  |
| CD | 0.45 |  |
| RMSEA, root mean squared error of approximation; AIC, Akaike’s information criterion; BIC, Bayesian information criterion; CFI, comparative fit index; TLI, Tucker–Lewis index; SRMR, standardized root mean squared residual; CD, coefficient of determination | | |
|  | | |
|  | | |
|  | | |
|  | | |
|  | | |
|  | | |
|  | | |
